# *FAD2-DGAT2* Genes Coexpressed in Endophytic *Aspergillus fumigatus* Derived from Tung Oilseeds

**DOI:** 10.1100/2012/390672

**Published:** 2012-07-31

**Authors:** Yi-Cun Chen, Yang-Dong Wang, Qin-Qin Cui, Zhi-Yong Zhan

**Affiliations:** ^1^State key Laboratory of Forest Genetics and Tree Breeding, Chinese Academy of Forestry, Beijing 100091, China; ^2^Institute of Subtropical Forestry, Chinese Academy of Forestry, Fuyang 311400, China

## Abstract

Recent efforts to genetically engineer plants that contain fatty acid desaturases to produce valuable fatty acids have made only modest progress. Diacylglycerol acyltransferase 2 (DGAT2), which catalyzes the final step in triacylglycerol (TAG) assembly, might potentially regulate the biosynthesis of desired fatty acids in TAGs. To study the effects of tung tree (*Vernicia fordii*) *vfDGAT2* in channeling the desired fatty acids into TAG, *vfDGAT2* combined with the tung tree fatty acid desaturase-2 (*vfFAD2*) gene was co-introduced into *Aspergillus fumigatus*, an endophytic fungus isolated from healthy tung oilseed. Two transformants coexpressing *vfFAD2* and *vfDGAT2* showed a more than 6-fold increase in linoleic acid production compared to the original *A. fumigatus* strain, while a nearly 2-fold increase was found in the transformant expressing only *vfFAD2*. Our data suggest that *vfDGAT2* plays a pivotal role in promoting linoleic acid accumulation in TAGs. This holds great promise for further genetic engineering aimed at producing valuable fatty acids.

## 1. Introduction

Various forms of Δ^12^-oleic acid desaturase (FAD2), which catalyzes the introduction of a cis-Δ^12^ double bond into oleic acid to produce linoleic acid, have recently been used in genetically engineered plants [1]. FAD2-like enzymes are able to produce fatty acids, but they are not effective at channeling fatty acid removal from phosphatidylcholine and their accumulation as triacylglycerols (TAGs) [2, 3]. Diacylglycerol acyltransferase 2, which catalyzes the final step of TAG biosynthesis in the classical Kennedy pathway, might mediate the accumulation of the desired fatty acids in TAGs [2]. For example, *Ricinus communis* fatty acid hydroxylase 12 (FAH12) produced nearly 30% more hydroxy fatty acid when coexpressed with *R. communis *type-2 acyl-coenzyme A: diacylglycerol acyltransferase (RcDGAT2) [4]. There is evidence that tung tree (*Vernicia fordii*) *vfDGAT2* enhances trieleostearic biosynthesis in yeast [5]. We examined vfDGAT2 function in the incorporation of linoleic acid into TAGs in a transgenic fungus.

## 2. Materials and Methods

### 2.1. Materials

Nuts were collected from tung oil trees in Tianlin County, Guangxi Zhuang Autonomous Region, China. Healthy nuts were harvested during the developmental stage on 10 September, when tung oil accumulates during seed maturation [5]. The nuts were hand-shelled and the kernels were frozen immediately in liquid N_2_ and then stored at –80°C. The reagents used were purchased from Takara, Promega, and Invitrogen. Oligonucleotides were synthesized by Sangon Biotech. DNA was isolated and purified using a kit from Axygen. Plant clone vector pMD18-T was purchased from Takara. Plant expression vector pCAMBIA1301 was preserved in our lab.

### 2.2. **Aspergillus fumigatus ** Derived from Tung Oilseeds

The endophytic fungus *A*.* fumigatus* was isolated from oilseeds of healthy tung trees and cultivated on yeast extract peptone dextrose agar (YPD). The strain was identified based on its morphological characteristics and molecular data (nuclear ribosomal internal transcribed spacer sequence).

### 2.3. Genes Isolated from Tung Oilseeds

Total RNA was extracted from tung kernels using an improved TRIzol method. cDNA was obtained by reverse transcription and used as the template for polymerase chain reaction (PCR). Specific primers were designed from sequences in GenBank (*vfFAD2*, accession number AF525534; *vfDGAT2*, accession number DQ356682) (Table 1).

### 2.4. Cointroduction of *vfFAD2-vfDGAT2* into *A. fumigatus* Using Agrobacterium Tumefaciens-Mediated Transformation

**Figure 1:**
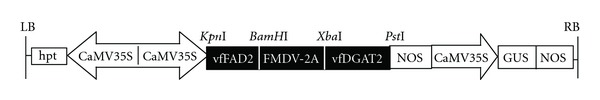
Construction of *vfFAD2-vfDGAT2/*pCAMBIA1301 using the *FMDV2A* sequence. LB: the left T-DNA border; hpt: hygromycin gene; CaMV35S: cauliflower mosaic virus 35S promoter; *vfFAD2*: tung tree; linoleic acid desaturase gene *FMDV2A*: foot-and-mouth disease Virus 2A sequence; *vfDGAT2*: diacylglycerol acyltransferase gene; NOS: NOS terminator; GUS: beta-glucuronidase; RB: the right T-DNA border.

The foot-and-mouth disease virus 2A (FMDV-2A) sequence, which cleaves proteases, was used to conjugate *vfFAD2 *with* vfDGAT2*. The three sequences were subcloned into expression vector pCAMBIA1301 using different restriction enzymes (Figure 1, Table 1). The recombinant expression vector contained a unique open reading frame (ORF) for *vfFAD2*-*FMDV2A*-*vfDGAT2*, with an initiation codon at the beginning of the *vfFAD2 *sequence and a stop codon at the end of the *vfDGAT2 *sequence (Table 1).

*Agrobacterium tumefaciens*-mediated transformation was used to transform *vfFAD2*-*FMDV2A*-*vfDGAT2*/*2/*pCAMBIA1301 into *A*.* fumigatus*. The transformants were first selected on selection medium (SM) plates using hygromycin B (250 *μ*g/mL), cefotaxime (400 *μ*g/mL), and streptomycin (60 *μ*g/mL). Then the obtained transformants were further selected on an SM plate containing 100 *μ*g/mL hygromycin B. A PCR method was used to identify whether the genes were integrated into the *A*.* fumigatus* chromosomesuccessfully using the primers listed in Table 1.

### 2.5. Fatty Acid Analysis****

The crude oil was obtained by soxhlet extraction. To analyze the fatty acid composition, fatty acid methyl esters (FAMEs) were prepared using potassium methoxide [6]. The FAMEs were purified using an AccuBOND solid-phase extraction column and evaporated to dryness under a nitrogen stream. The residue was resuspended in 1 mL 10% isopropanol in* n*-hexane containing methyl heptadecanoate as an internal standard.

Gas chromatography was performed on a Hewlett-Packard 5890 series II gas chromatograph (Agilent) equipped with flame ionization detection (FID) and a Supelco SP-2380 column (Sigma-Aldrich). Helium was used as the carrier gas at a flow rate of 8 mL/min. The inlet and detector were held at 200°C. The temperature of the column oven was programmed to increase from 110°C to 160°C at a rate of 17°C/min, then to 175°C at 5°C/min, and finally to 200°C at 3°C/min, and held for 3 min. The data were collected and processed using a Hewlett-Packard 3365 series II ChemStation.

## 3. Results and Discussion

### 3.1. **vfFAD2/vfDGAT2 ** Coexpressed in **A. fumigatus**

**Figure 2:**
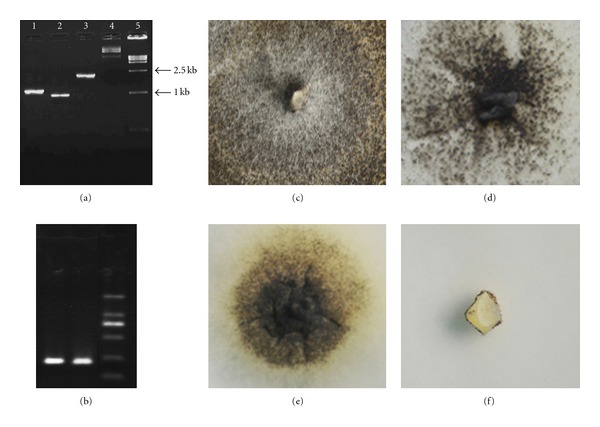
PCR verification of the *vfFAD2-FDMV2A-vfDGAT2*/pCAMBIA1301 recombinant vectors and *A. fumigatus*/*vfFAD2-FDMV2A-vfDGAT2* transformant. (a)PCR verification of the *vfFAD2-FDMV2A-vfDGAT2*/pCAMBIA1301 recombinant vector. (b) PCR amplification of the *A. fumigatus* transformant using special primers. (c–f) *A. fumigatus* transformants grew in PDA medium containing 100 *μ*g/mL hygromycin B. 1, *vfDGAT2; 2: vfFAD2; 3: vfFAD2-FMDV2A-vfDGAT2*; 4: pCAMBIA1301 vector; 5: DNA Marker. (c) *A*. *fumigatus*/*vfFAD2-FDMV2A-vfDGAT2*; (d)*A.   fumigatus*/*vfFAD2*: (e) *A. fumigatus*/*vfDGAT2*; (f) *A. fumigatus*.

The ORF sequence of the tung linoleic acid desaturase gene (*vfFAD2*) was 1152 base pairs (bp) long and that of tung diacylglycerol acyltransferase gene (*vfDGAT2*) was 968 bp (Figure 2(a)). The *vfFAD2-FDMV2A-vfDGAT2/*pCAMBIA1301 recombinant vector was constructed successfully (Figures 1 and Figure 2(a)). The transformants of *A*. *fumigatus/vfFAD2*, *A*.* fumigatus/vfDGAT2*, and *A*.* fumigatus/vfFAD2-FDMV2A-vfDGAT2* are shown in Figures 2(b)–2(f). Specific primers for *vfFAD2 *and* vfDGAT2* were used to identify the transformants using PCR (Table 1).

### 3.2. Fatty Acid Profile in the **A. fumigatus ** Transformants

**Figure 3:**
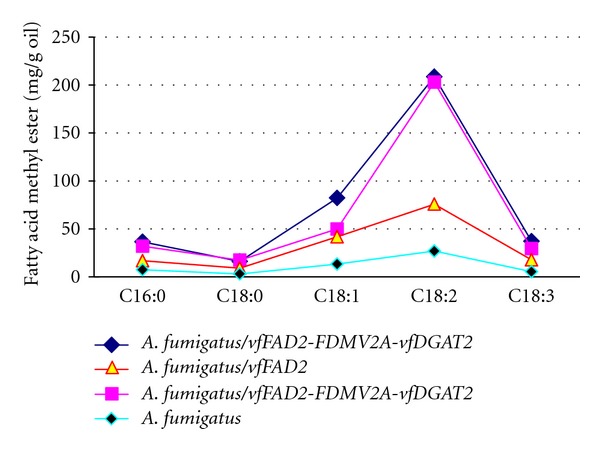
The fatty acid profile of *A*. *fumigatus *transformants containing* vfFAD2 *and *vfDGAT2*. C16 : 0, palmic acid; C18 : 0, stearic acid; C18 : 1, oleic acid; C18 : 2, linoleic acid; C18 : 3, linolenic acid. The two *A*.* fumigatus* transformants with *vfFAD2-FDMV2A-vfDGAT2 *showed a more than sixfold increase in linoleic acid production, while the transformant expressing *vfFAD2* had about a twofold increase compared to the original *A. fumigatus* strain.

Fatty acid production in *A*.* fumigatus* was investigated using Gas chromatography. Five fatty acids were identified: palmic acid (C16 : 0), stearic acid (C18 : 0), oleic acid (C18 : 1), linoleic acid (C18 : 2), and linolenic acid (C18 : 3). Because the *A*.* fumigatus* transformant with *vfDGAT2* grew so slowly, its fatty acids were not considered. The linoleic acid production in *A*.* fumigatus* expressing only* vfFAD2 *was 1.81 times that in *A*.* fumigatus*. In contrast, that in *A*.* fumigatus* transformed with* vfFAD2-FDMV2A-vfDGAT2* was more than 6 times that of the control (Figure 3). Fatty acid desaturase 2 (vfFAD2) in tung oilseed catalyzes the conversion of oleic acid (C18 : 1) into linoleic acid (C18 : 2) [7]. Nevertheless, linoleic acid production increased only modestly in* A*.* fumigatus* expressing the *vfFAD2 *gene only. Diacylglycerol acyltransferase (vfDGAT2) catalyzes the final step in TAG biosynthesis, and it likely improves the fatty acid accumulation when coexpressed with *vfFAD2*.

Recent efforts to genetically engineer plants to produce unusual fatty acids have been met with only modest success, because the fatty acids flux into substrate pools instead of TAG [2]. This study used a seed-associated endophytic fungus as the transformation model. Consequently, the fatty acid desaturase was able to catalyze linoleic acid production, and *vfDGAT2* seemed to play a role in prompting fatty acid flux into TAG in the transgenic fungus. Therefore, *vfDGAT2* might be a candidate gene mediating fatty acid assembly into TAG, which holds promise for transgenic engineering aimed at producing valuable fatty acids.

## Figures and Tables

**Table 1:** Primer sequences of *vfFAD2, vfDGAT2*, and *FDMV*-2*A*.

	Primers	Sequences 5^′^-3^′^
1	*vfFAD2* Forward (*Kpn*I)	GGc**ATG**GGTGCTGGTGGC
2	*vfFAD2* Reverse (*Bam*HI)	CGggatccAAACTTCTTGTTATACC
3	*FD* *MV*-2*A* Forward (*Bam*HI)	gatccCAGCTGTTGAATTTTGACCTTCTTAAGCTTG CGGGAGACGTCGAGTCCAACCCCGGGT
4	*FD* *MV*-2*A* Reverse (*Xba*I)	gCCCGGGGTTGGACTCGACGTCTCCCGCAAGCTT AAGAAGGTCAAAATTCAACAGCTGAGATC
5	*vfDGAT2* Forward (*Xba*I)	GCtctagaATGGGGATGGTGGAAGTTAAG
6	*vfDGAT2* Reverse (*Pst*I)	AActgcag**TCA**AAAAATTTCAAGTTTAAGG
7	*vfFAD2* Forward^′^	CGCCGTCACCACTCTAAC
8	*vfFAD2 *Reverse^′^	AGGCCAGCCAAGGGTAAGTGT
9	*vfDGAT2* Forward^′^	GGGGATGGTGGAAGTTAAGAA
10	*vfDGAT2 *Reverse^′^	AAGCAACCCAATAACGAGGAG

The nucleotides in lowercase letters indicate restriction sites. ATG and TCA in bold indicate the initiation and stop codon, respectively, for the open reading frame of *vfFAD*2-*FMDV*2*A*-*vfDGAT*2. Primers 7–10 were used to amplify *vfFAD*2 and *vfDGAT*2to give PCR products of 150 and 190 bp, respectively.
